# A simple approach for multi-targeted shRNA-mediated inducible knockdowns using *Sleeping Beauty* vectors

**DOI:** 10.1371/journal.pone.0205585

**Published:** 2018-10-19

**Authors:** Severin Fink, Laurens Zugelder, Bernhard Roth, Evelyn Brandt, Sylvain Meloche, Zsuzsanna Izsvák, Ralf C. Bargou, Thorsten Stühmer

**Affiliations:** 1 Comprehensive Cancer Center Mainfranken, University Hospital of Würzburg, Würzburg, Germany; 2 Institute for Research in Immunology and Cancer, University of Montreal, Montreal, Canada; 3 Max Delbrück Center for Molecular Medicine in the Helmholtz Association, Berlin, Germany; Friedrich-Loeffler-Institute, GERMANY

## Abstract

shRNA expression is an established technique to transiently or permanently deplete cells of a particular mRNA/protein. In functional analyses of oncogenic pathways it can thus be used as an alternative to pharmacologic inhibitors, or as a means to address otherwise undruggable targets. Here we describe and functionally validate a simple reiterative cloning system to generate concatenated multi-shRNA expression plasmids. The multi-shRNA expression cassette can eventually be subcloned into any suitably designed vector for the stable transfection of cells, here tested with derivatives of the *Sleeping Beauty* transposon vector for stable transfection of multiple myeloma cell lines at the lowest biosafety level. We finally test inducible versions of such multi-cassette knockdown vectors and show their efficacy for the induced concerted knockdown of all four components of the MEK/MAPK-module in the Ras/MAPK pathway. The described vector system(s) should be useful for functional knockdown analyses in a wide array of cell line models.

## Introduction

RNAi approaches to temporarily or permanently deplete cells of a selected mRNA/protein have become a commonplace molecular procedure in functional analyses [1]. They can provide an independent control to validate the potential effects of small molecule inhibitors or other types of drug, and they offer the possibility to investigate targets that have so far been undruggable. The two main forms of RNAi treatment of cells in culture are delivery of short *in vitro* synthesized double-stranded RNA-oligonucleotides (siRNA), and introduction of vectors for expression cassettes of short-hairpin RNAs (shRNA), in which case the cell itself will transcribe the shRNA gene and further proceed the product to a functional dsRNA oligonucleotide [2, 3]. siRNAs need to be expensively bought as reagents, but their small size and ready-to-use design for the cell's RNA-induced silencing complex make them very useful tools for efficient delivery to target cells in transient knockdown analyses. shRNA vectors on the other hand are bulky reagents, but–once constructed–they are easily produced and shared, and they can be used in transient as well as in stable knockdown experiments. Additionally, these systems can be designed to be inducible, potentially avoiding negative selection effects during establishment of stably transfected cultures [4]. The choice of any particular RNAi approach may therefore be influenced by many factors, such as availability of reagents, the particular cell line and the desired short- or long-term functional readouts.

Contrary to invertebrate species, vertebrate signal transduction pathways often encode multiple paralogous isoforms of core components that act in a functionally redundant manner. This is well exemplified by the ERK1/2 MAP kinase signaling pathway, which is composed of two MEK and ERK isoforms [5]. Thus, genetic inactivation of these signaling pathways requires the simultaneous targeting of multiple genes. Also, cancer cells often rely on oncogenic signaling networks, and thus on combinations of pro-proliferative and anti-apoptotic signals [6, 7]. In order to analyze which pathways need to be targeted in combination therapies for efficient cell death it is therefore advantageous if shRNA approaches can be conducted via a single vector that addresses multiple targets. Here, we have chosen the pSUPER-based shRNA expression system [8–10] and modified it in such a way, that "any" number of the same or of different shRNA expression cassettes can easily be assembled within a single vector. The same subcloning procedure can subsequently be used to transfer the complete multi-cassette shRNA expression construct into a modified version of the *Sleeping Beauty* (pT2) transposon vector for stable transfection of mammalian cells [11]. Here, we test the applicability and efficiency of the system in its constitutively active and doxycyclin-inducible forms by collectively knocking down the four components of the MEK/MAPK module (the proteins MAP2K1 (MEK1), MAP2K2 (MEK2), MAPK3 (ERK1), and MAPK1 (ERK2)) in transient and stable transfections of multiple myeloma cell lines.

## Materials and methods

### Human MM cell lines and cell culture

MM cell lines were either bought from the German Collection of Microorganisms and Cell Cultures (DSMZ, Braunschweig, Germany) (AMO-1, JJN-3, L-363, OPM-2) or from LGC Standards (Wesel, Germany) (MM.1s). INA-6 cells [12] were obtained from Martin Gramatzki (University Medical Center Schleswig-Holstein, Kiel, Germany). New cell lines were immediately expanded into stock and working banks, checked for absence of mycoplasma [13] and stored in liquid nitrogen. Cell cultures were freshly reinstated from working bank aliquots every 3–4 months (dead-end culture), thus assuring authenticity. Cell culture was performed at 5% CO_2_ and 37°C in RPMI-1640 medium supplemented with 10% FBS, 1 mM sodium pyruvate, 2 mM glutamine, 100 U/ml penicillin, and 100 μg/ml streptomycin. INA6 cells were supplied with 2 ng/ml recombinant human interleukin-6.

### shRNA sequences and reagents

The target sequences for human ERK1 (5'-GCCATGAGAGATGTCTACA; cDNA positions 340–358) and ERK2 (5'-GAGGATTGAAGTAGAACAG; cDNA positions 900–918) have been described and functionally validated before [14]. The human MEK1 target sequence was based on a sequence taken from the database of the BROAD Institute (5'-GAGGGAGAAGCACAAGATCA; cDNA positions 612–631; TRCN0000002331), and the one finally chosen for MEK2 (5'-GAAGGAGAGCCTCACAGCA; cDNA positions 862–880) was based on an siRNA sequence published in [15]. The MEK1&2 inhibitor PD0325902 was bought from Selleck Chemicals, München, Germany (S1036).

### Construction of a pT2-based EGFP-expression vector

Vector pEGFP-N3 (Clontech) was digested with *Bgl* II and *Bam* HI and religated, which removes most of the multiple cloning site, including the single *Eco* RI site. Subsequently, two *Eco* RI sites flanking the complete CMV promotor-driven EGFP expression cassette were introduced by site-specific mutagenesis using the QuikChange protocol, and this cassette was subcloned into the single *Eco* RI site of the *Sleeping Beauty* vector pT2-SVneo, yielding vector pT2-SVneo-CMV-EGFP.

### Electroporation of MM cells and establishment of stably transfected MM cell cultures

The protocol for MM cell electroporation with expression vectors for shRNA or protein templates and the selective purification of strongly transfected viable cells for Western blotting is described in detail in [16]. The standard plasmid concentration used in electroporations was 20 μg/ml for each pSUPER- and pT2-based expression vector, and 30 μg/ml for the transposase expression plasmid pCMV-SB100X [17]. 5 μg/ml pEGFP-N3 was included in electroporation mixtures to assess transfection efficiency. Selection reagents (G418 at 0,5–1 mg/ml and/or puromycin at 2 μg/ml) were added to electroporated cultures at day 2 post-transfection. After about 7 days the culture was spun down, resuspended in fresh full medium with selection reagents and further incubated until a stably transfected culture was established (2–4 weeks, depending on the transfection and growth properties of the different MM cell lines).

### Western blotting and antibodies

The protocol for Western blotting is described in detail in [18]. The following antibodies were used: anti-ERK1,2 (Santa Cruz Biotechnology, Heidelberg, Germany; sc-94), anti-phospho-ERK1,2 (Cell Signaling Technology (CST), Frankfurt am Main, Germany; no. 9101), anti-MEK1,2 (CST; no. 9122), anti-phospho-MEK1,2 (CST; no. 9154), anti-HA-tag (Abcam, Cambridge, UK; ab9110), anti-tet-Repressor (Merck, Darmstadt, Germany; AB3541), anti-HSP90β (Enzo Life Sciences, Lörrach, Germany; ADI-SPA-843), anti-α-tubulin (Bio-Rad, München, Germany; MCA78G). Secondary antibody F(ab')2 fragments coupled to horseradish peroxidase and specific for rabbit-IgG (no. 111-036-045), mouse-IgG (no. 115-036-072) or rat (no. 112-036-062) were obtained from Jackson ImmunoResearch, Newmarket, UK.

A freshly made solution of luminol (2.5 mM), p-coumaric acid (0.2 mM) and H_2_O_2_ (0.01%) in 100 mM Tris-HCl (pH 8.8) was used as reagent for chemiluminescent detection [19].

## Results and Discussion

### Rationale

In order to achieve the final goal: an easy-to-assemble and functionally well applicable inducible system for the stable and simultaneous specific knockdown of multiple targets, we tried to modify the pSUPER-shRNA expression system [8], which we had previously been using for knockdown experiments in MM cells [14, 16], and to combine it with *Sleeping Beauty*-mediated stable transposition [11]. The pT2 *Sleeping Beauty* transposon-based vectors are attractive for stable integration into human cells because they are easy to use and they require only the lowest biosafety level. In order to prevent selection bias or to even permit the growth of stable integrants that functionally express detrimental shRNAs, tight repression and good inducibility are both essential elements. In addition, the system was intended to permit the unconstrained assembly of any desired combination of shRNA expression cassettes within a single transposable vector, and to allow a prediction of the expected knockdown efficacy based on the results of transient single-target knockdown with pSUPER.

### Construction of a multi-target vector system for transient knockdown

**Fig 1:**
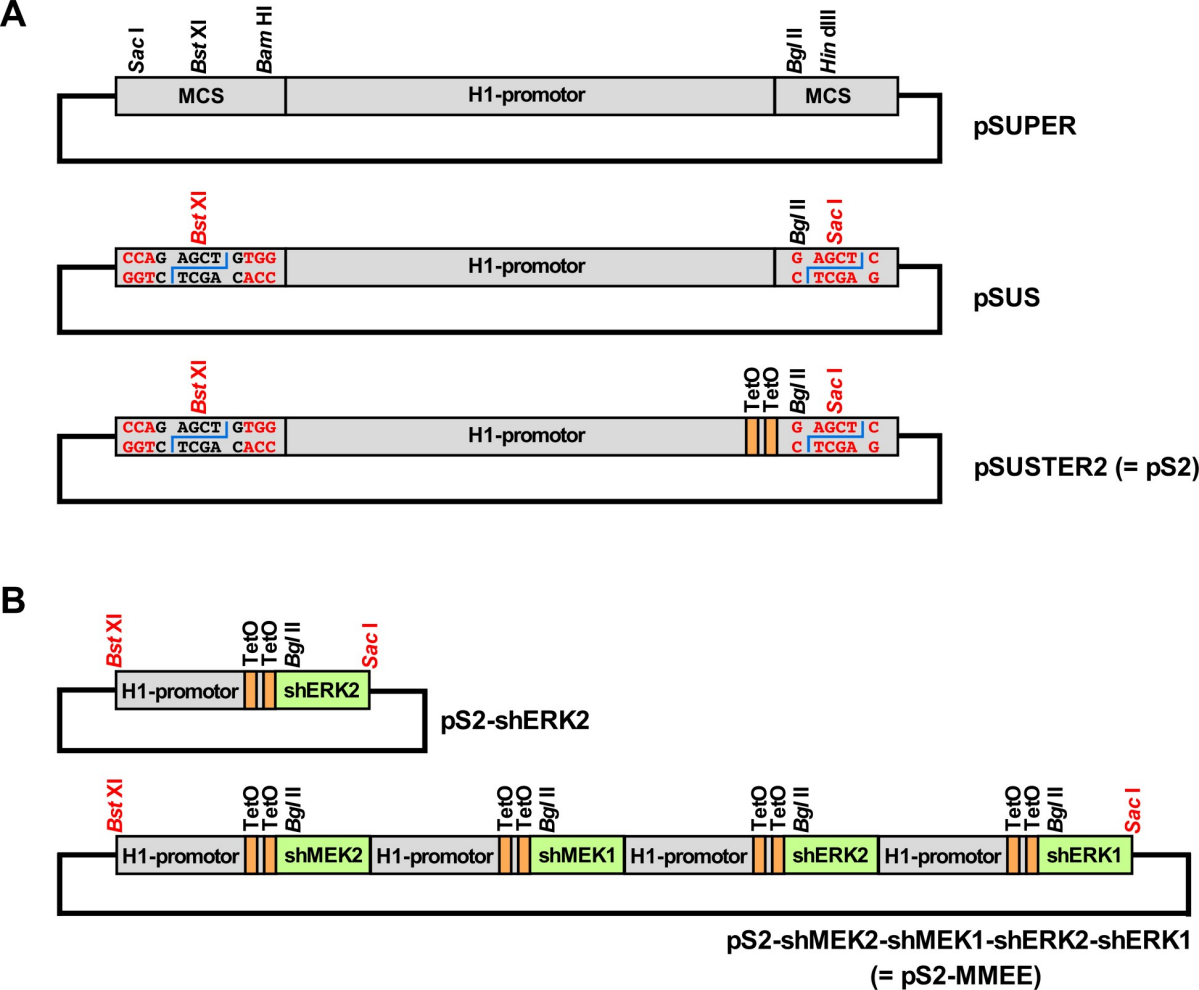
Schematic representations of the major steps from pSUPER to concatenable and regulatable multi-shRNA-expression vectors. (A) pSUPER. A sequence encoding a specific shRNA would be introduced using the *Bgl* II and *Hin* dIII sites. Middle: **pSU**PER-***S****ac* I (= pSUS). A sequence encoding a specific shRNA would be introduced using the *Bgl* II and the newly generated *Sac* I site (in red). Upstream of the H1 promoter the formerly present *Sac* I site has been destroyed, and the original *Bst* XI site has been modified in such a way that the 6 optional core nucleotides represent a 3-prime-corrupted *Sac* I motif (GAGCT**G**) which is cleaved by *Bst* XI to generate *Sac* I compatible ends. Bottom: pSUSTER2 (= pS2). Two tet-operator (TetO) sequences [9, 10] within the H1 promoter yield the shRNA expression cassette doxycyclin-inducible in cells that constitutively express the tet repressor protein. (B) Versatile concatenability of the pS2-system. Top: Schematic representation of a pS2-series vector for expression of a single short hairpin RNA targeting the MAP kinase ERK2. Bottom: Schematic representation of a pS2-series vector containing 4 different inducible shRNA expression cassettes targeting MEK2, MEK1, ERK2 and ERK1. Head-to-tail subcloning of a *Bst* XI/*Sac* I-cut pS2 shRNA expression cassette into another pS2 vector linearized with *Bst* XI regenerates the 5'-*Bst* XI site but does not re-create any internal sites. Additional cassettes can thus be added *ad libitum*. For stable transfections the whole multi-shRNA expression cassette can be subcloned via *Bst* XI/*Sac* I-digest into any suitable vector that contains a unique *Sac* I compatible cloning site. MCS: multiple cloning site (of pBluescript KS(+), the parental plasmid of pSUPER).

We initially modified the pSUPER vector so that the concatenization of any desired number of similarly constructed shRNA expression cassettes becomes possible. In a first step, pSUPER was digested with *Sac* I and *Bam* HI, which removes most of the multiple cloning region that remains of pBluescript KS(+) (the parental plasmid of pSUPER) upstream of the H1 promoter (Fig 1A). Ligation of a ds oligonucleotide with compatible overhangs then led to two important changes: i) the single *Sac* I site in pSUPER is destroyed, and ii) a modified *Bst* XI site is now substituting for the original one. Both *Sac* I and *Bst* XI produce upon cleavage a 4 nucleotide 3'-overhang, and the recognition sequence of *Bst* XI (CCANNNNN^NTGG) contains a central 6 bp sequence of arbitrary nucleotides. By choosing CAGCTC (a 3-prime-corrupted *Sac* I recognition motif) as its core sequence the resulting *Bst* XI site therefore produces a *Sac* I compatible overhang upon cleavage, but ligation into this site does not generate a *Sac* I site.

In a second step, the *Hin* dIII site of the above-modified vector was changed into a *Sac* I site using the QuikChange site-directed mutagenesis kit. In the resulting plasmid (**pSU**PER-***S****ac* I = pSUS (Fig 1A)), the short-hairpin-defining ds oligonucleotide cloning is now performed via the *Bgl* II/*Sac* I sites, but the same functionality as for pSUPER vectors is expected. However, the complete shRNA expression cassette of a pSUS-vector can now be excised via *Bst* XI/*Sac* I digest and subcloned into the *Bst* XI site of any other pSUS-type vector. In the correct orientation this ligation regenerates the 5'-*Bst* XI site with its corrupted *Sac* I motif, but neither a *Sac* I site nor a *Bst* XI site are regenerated at the junction between both shRNA expression cassettes. This procedure can therefore be repeated as often as desired, and the thus generated concatenized shRNA expression cassette construct can itself be excised and further subcloned in the same way, because it always remains flanked by a 3' *Sac* I site and a 5' *Sac* I-compatible *Bst* XI site (Fig 1B). It is prudent, though, to use DNA analysis software to monitor the design of the individual shRNA expression constructs, since internal sites for *Sac* I, *Bgl* II and *Bst* XI must be avoided, and in unfortunate constellations such sites could even arise *de novo* from certain combinations of gene-specific and flanking sequences in the short hairpin design. Especially the *Bst* XI site can easily be overlooked.

**Fig 2:**
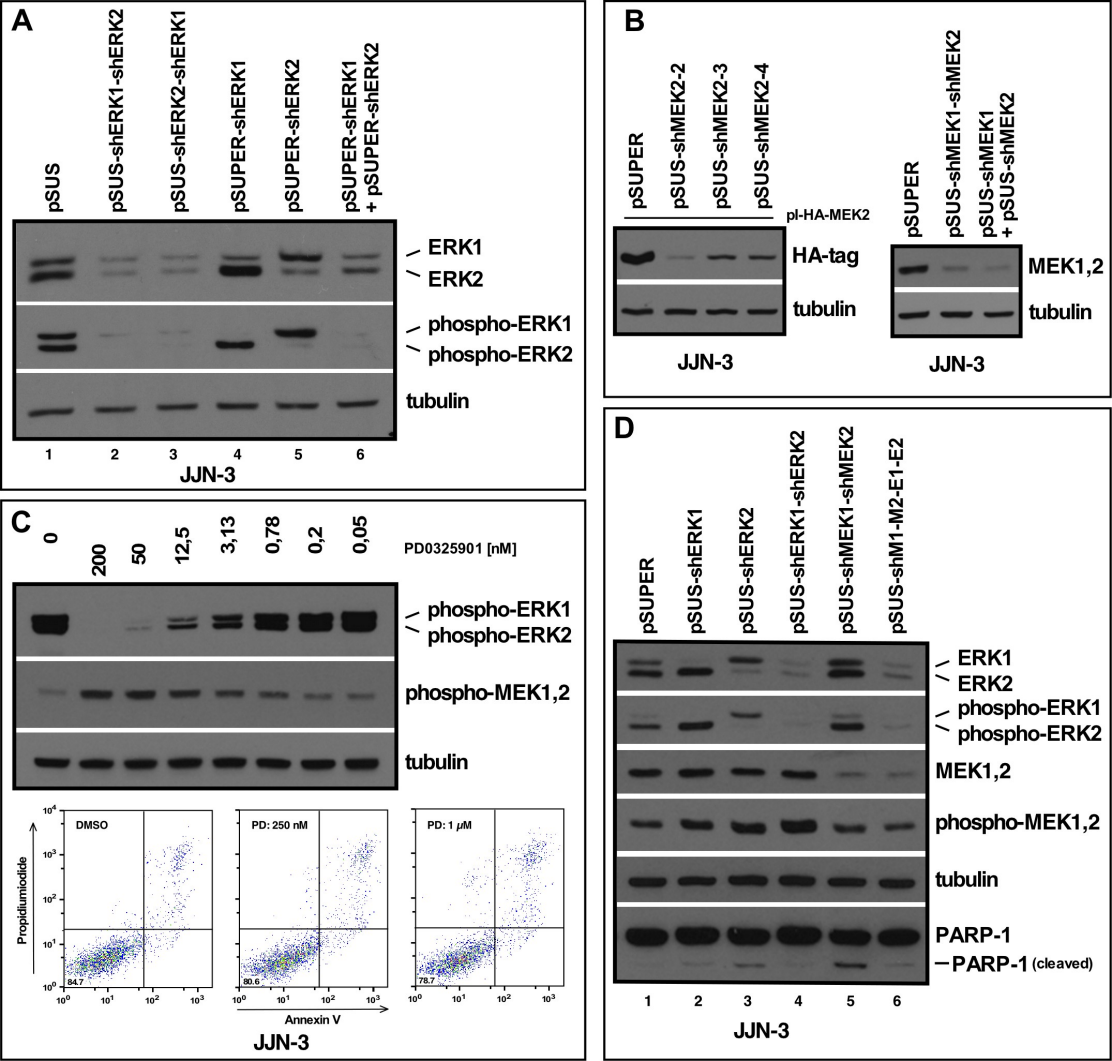
Establishment and characterization of vectors for concatenated shRNA expression cassettes. (A) Western blot showing the effects of selective ERK1 and/or ERK2 knockdown in JJN-3 MM cells. Cells were transfected with plasmid solutions by electroporation, and subsequently purified based on the presence of CD4Δ (see Methods and [8]). Cells were then harvested for Western blotting at day three post-transfection. Knockdown of both ERK1 and ERK2 from the pSUS double-cassette vectors (lanes 2 and 3) matches the effect obtained with the pSUPER-based single-cassette vectors (lanes 4–6). (B) Left: Establishment of knockdown constructs for MEK2 (three different target sequences tried), based on lowered expression of HA-tagged human MEK2 from a co-transfected expression plasmid (see Methods). The shMEK2-2 sequence was chosen for further experiments (= shMEK2). Right: Knockdown of MEK1 and MEK2 from a pSUS double cassette vector (middle lane) versus combined transfection of the single shRNA expression vectors (right lane). (C) Concentration-dependent effects of the MEK1&2 inhibitor PD0325906 on the levels of phospho-ERK1/2 and phospho-MEK1/2. Cells were treated for 1 h with the drug and harvested for Western blotting. Substantial downregulation of phospho-ERK1/2 results in inceased levels of phospho-MEK1/2. FACS analysis with staining for early (annexin V) and late (PI) apoptotic markers after 3 days of drug treatment does not show increased rates of apoptosis. (D) Western analysis of the efficiency of MEK1+2 and ERK1+2 knockdown from a quadruple cassette shRNA-expression vector (lane 6) in relation to single cassette vectors (ERK1, ERK2, lanes 2 and 3) and double cassette vectors (ERK1+2, lane 4; MEK1+2, lane 5). Cells were harvested at day 3 post-transfection.

In order to compare the knockdown efficacy of such multiple cassette vectors, in which each shRNA coding sequence has its own (and identical) promotor, to that of single-cassette vectors, we chose the MEK1/2-ERK1/2 module of the Ras-MAPK-pathway as a suitable target for a quadruple knockdown. Very efficient blockade of this pathway can be achieved in multiple myeloma (MM) cells with the small molecule allosteric MEK1/2 inhibitor PD0325901 (Fig 2C), but although such treatment can attenuate proliferation of MM cells, it does not generally induce cell death ([14], [20], Fig 2C). Furthermore, once MEK1/2 blockade leads to substantial abrogation of the downstream phospho-ERK signal, feedback mechanisms entail increased phosphorylation of MEK1/2 themselves, thus providing a useful readout for the functional blockade of the pathway (Fig 2C).

We initially constructed individual pSUS-constructs for shERK1 and shERK2, because we had successfully been using the respective target sequences in pSUPER [13] and from these constructed the double cassette vectors pSUS-shERK1>shERK2 and pSUS-shERK2>shERK1. The plasmids were transfected into JJN-3 MM cells by electroporation and the most strongly transfected cells were purified by MACS selection (see Methods and [16]) and harvested for Western blotting at day 3 post-electroporation. Cells treated likewise with empty pSUS vector, and with pSUPER-shERK1 and/or pSUPER-shERK2 vectors served as benchmark controls. ERK1/2 knockdown efficacy was found to be on a par with the pSUPER-system and no positional effect was discernible for the double-constructs, i.e. knockdown efficiency of either ERK1 or ERK2 as well as decrease of the respective phospho-ERK levels was independent on the relative position of the shRNA expression cassettes (Fig 2A).

Next, we developed vectors pSUS-shMEK1 and pSUS-shMEK2 by testing various candidate sequences derived from the database of the BROAD Institute or from published sequences. Knockdown efficacy was checked in JJN-3 cells transiently transfected with the respective single shRNA expression vectors and co-transfected with expression plasmids for human HA-tagged-MEK1 (not shown) or human HA-tagged-MEK2 (Fig 2B, left) because the MEK proteins are indistinguishable on PAA gels. A pSUS-shMEK1>shMEK2 double cassette vector was subsequently constructed and MEK1/2 knockdown again assessed in transiently transfected JJN-3 cells. Again, the double knockdown vector was found to be as effective as a combination of both single expression vectors (Fig 2B, right). Finally, this shMEK1>shMEK2 cassette was subcloned into the shERK1>shERK2 expression plasmid, and the quadruple vector (in short: pSUS-shM1>M2>E1>E2) compared for its knockdown efficiency of the complete MEK1/2-ERK1/2 module against the single ERK, double ERK and double MEK knockdown vectors (Fig 2D). No decrease in knockdown efficiency when compared against either the single ERK or the double ERK or MEK vectors was visible in cells harvested on day 3 post-electroporation (Fig 2D). Of note, increased phospho-MEK1/2 levels in either the shERK1>shERK2 double knockdown construct, or the double MEK and quadruple constructs (the latter two show essentially identical phospho-MEK levels as the pSUPER control, but do so at much lower levels of MEK1 and 2 proteins (Fig 2D)) attested to the functional downregulation of the MAPK pathway. Virtually unchanged cleavage of PARP-1 indicated that these knockdowns had no significant effect on apoptosis induction of the transfected cells (Fig 2D).

### Stable transposition of MM cells with *Sleeping Beauty* vectors

**Fig 3:**
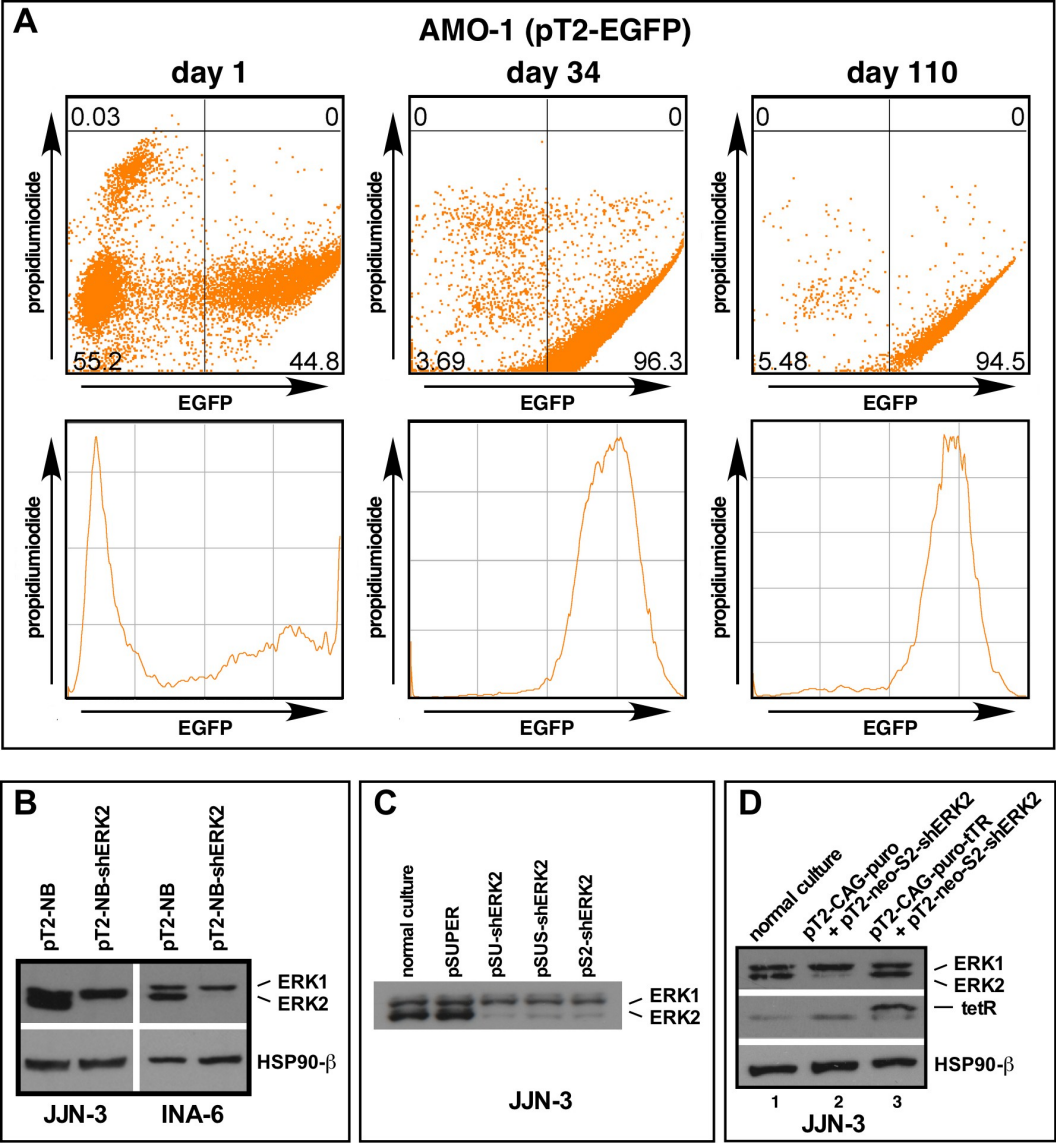
Establishment of transposon-mediated stable and regulatable shRNA expression in myeloma cells. (A) Stable transfection of AMO-1 MM cells with an EGFP-expressing *Sleeping Beauty* vector. Shown are FACS analyses of the whole culture at day 1 post-electroporation (left), and–following 2 weeks of selection with G418 –at days 34 (middle) and 110 (right) post-electroporation (see Methods for a detailed description). (B) Stable constitutive knockdown of ERK2 in wild-type JJN-3 and INA-6 MM cells after transfection with a *Sleeping Beauty* (pT2) vector containing an shRNA-expression cassette targeting ERK2. Western analysis of cells harvested at day 21 post-electroporation. (C) Western analysis of ERK2 knockdown efficiency from pSUPER-shERK2 and its derivatives in transiently transfected JJN-3 cells. (D) Repression of a transposed shERK2 expression cassette in JJN3 cells that stably express the tet-repressor protein (lane 3 vs. lane 2).

We next evaluated the utility of *Sleeping Beauty* transposon vectors for the stable expression of genes introduced into MM cells. The CMV promotor-driven EGFP expression cassette from vector pN3 was subcloned into *Sleeping Beauty* ((pT2); see Methods) and this vector (pT2-EGFP)–in combination with an SB100X transposase expression plasmid–electroporated into MM cells [16, 17]. G418 selection led to outgrowth of homogenously green cultures within 2-4 weeks, and further maintenance in culture was performed without geneticin. EGFP expression remained stable and its intensity virtually unchanged for at least 6 months, at which time the cultures were retired (tested for MM cell lines AMO-1 and INA-6; an initial FACS-analysis at day 1 post-electroporation and at two (much) later timepoints after G418 selection are shown for AMO-1 cells in Fig 3A). This system therefore provides a fast and convenient way to generate stably transfected polyclonal MM cell cultures at the lowest biosafety provisions. We then introduced the *Sac* I-compatible *Bst* XI site into the pT2neo vector (plasmid "pT2-NBneo"), subcloned the shERK2 expression cassette into this vector and analyzed the efficacy of stable ERK2 knockdown after transposition and selection as described above. Robust and specific loss of ERK2 was observed in all MM cell lines thus tested (Fig 3B; also performed for L-363 and AMO-1), although the effect may wear off somewhat upon long-term culture (in excess of 3 months) in some MM cell lines (L-363, not shown). These experiments showed that the pT2 vectors are excellent mediators of short and medium-term (at least 3–5 months) constitutive downregulation of target genes in MM cell lines.

### Inducible single- and multi-target knockdown in MM cells

In order to obtain inducible shRNA expression we introduced two tet-operator (tetO) sequences exactly as previously described [9, 10] into the H1 promotor region of pSUS, generating plasmid pSUSTER2 (= pS2; Fig 1A), a modification that had no effect on ERK2 knockdown efficiency when compared with pSUPER-based constructs in transiently transfected cells (Fig 3C). We then stably expressed the tet-repressor (tetR) protein in MM cell lines, using a modified *Sleeping Beauty* vector with a CAG-promotor-driven expression cassette and also harbouring a puromycin resistance gene. This system led to detectable levels of tetR protein in JJN-3 cells (Fig 3D, lane 3), and–after a second round of *Sleeping Beauty*-mediated transposition and double selection with G418 and puromycin–achieved suffient repression of a 2x tetO-sequence-containing shERK2-expression cassette to maintain ERK2 levels unchanged from those of normal cells in culture (Fig 3D, lanes 1 and 3).

**Fig 4:**
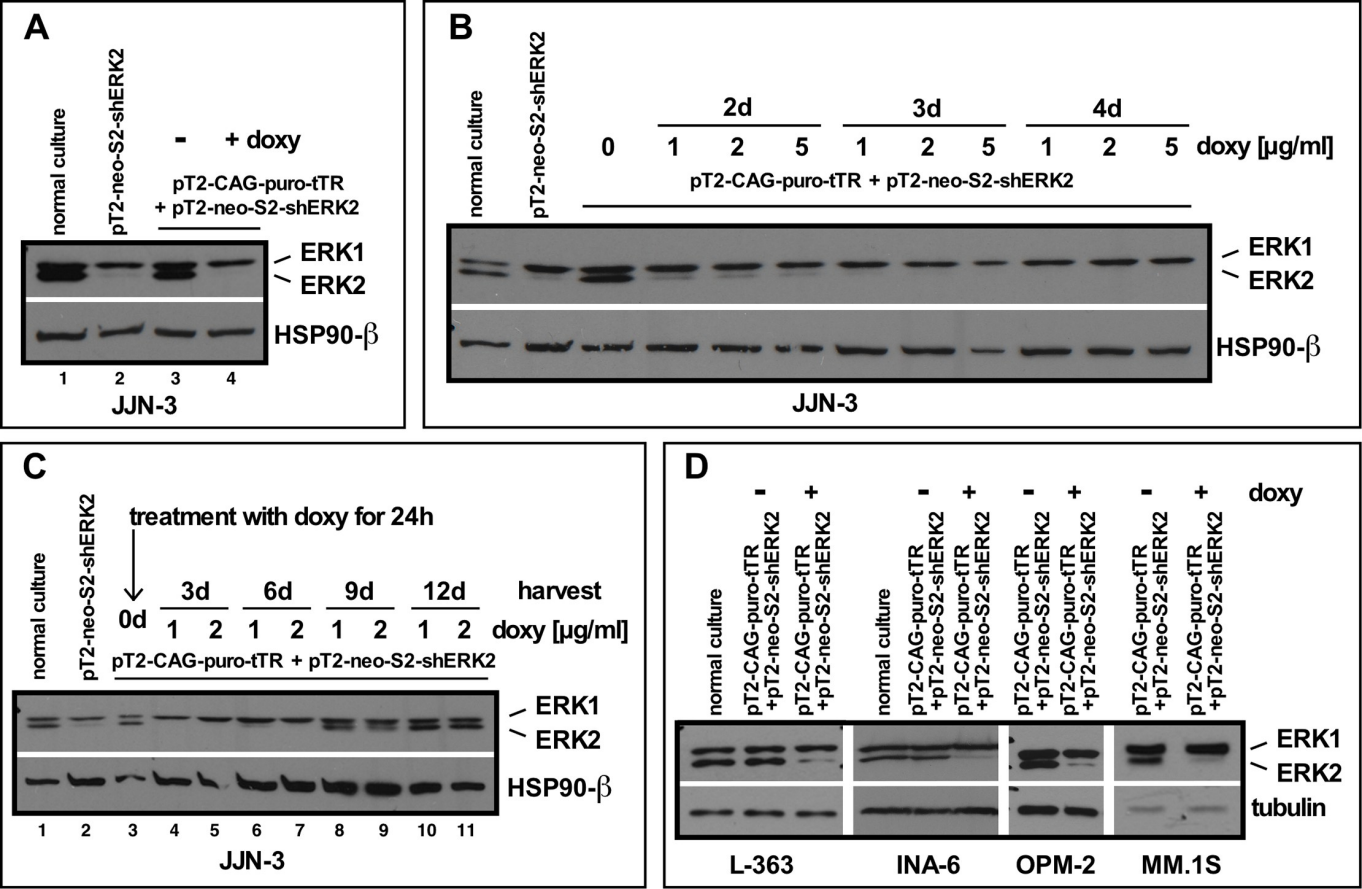
Establishment of inducible shRNA expression in MM cells. (A) ERK2 levels in JJN-3 cells stably transfected with the tet-repressor protein (pT2-CAG-puro-tTR) and an inducible shRNA cassette against ERK2 (pT2-neo-S2-shERK2) without (lane 3) and with 3 days of doxycyclin treatment (lane 4). Normal wildtype JJN-3 cells in culture (lane 1, basal ERK2 level) and after stable transfection with the pT2-neo-S2-shERK2 expression cassette (lane 2, ERK2 level in constitutive knockdown) serve as references. (B) Concentration- and time-dependence of doxycyclin-induced ERK2 knockdown in pT2-CAG-puro-tTR + pT2-neo-S2-shERK2 JJN-3 cells. Cells were treated with doxycyclin for 24 h. (C) Longer-term time course of induced ERK2 knockdown and ERK2 replenishment after 24 h of treatment of pT2-CAG-puro-tTR + pT2-neo-S2-shERK2 JJN-3 cells with either 1 or 2 μg/ml doxycyclin and subsequent culture for the indicated times. (D) Inducible ERK2 knockdown in different MM cell lines stably transfected with the pT2-CAG-puro-tTR + pT2-neo-S2-shERK2 combination after 3 days of treatment with 1μg/ml doxycyclin.

We used these cells to test the feasibility and efficacy of doxycyclin-mediated induction of ERK2 knockdown (Figs 4A–4C). Treatment with 2 μg/ml doxycyclin for 3 days entailed ERK2 depletion to levels found in cells not expressing the tetR-protein, i.e. under constitutive activity of the shERK2 expression cassette (Fig 4A, lane 4 vs. lane 2). We then tested concentration and time-dependence of doxycyclin-mediated ERK2 knockdown, and found no notable differences in knockdown efficiencies between concentrations of 1 and 5 μg/ml of doxycyclin (Fig 4B). For all concentrations strong knockdown was visible at day 2 post-induction, and after 3 days the ERK2 signal was barely detectable (Fig 4B). Lower concentrations of doxycyclin (0,5 or 0,25 μg/ml) led to greater variability in the ERK2 knockdown levels achieved (not shown), so either 1 or 2 μg/ml doxycyclin was chosen for further experiments. In order to test the reversibility of the inducible knockdown we treated the cells for 24 h with 2 concentrations of doxycycline (1 and 2 μg/ml) and then removed the drug with two washes in PBS. Cell culture was then continued without doxycycline and cells were harvested for Western analysis of ERK levels every 3 days. The 24 h doxycyclin pulse was sufficient to induce the maximal level of ERK2 depletion and this effect lasted for almost 6 days before the protein became gradually replenished (Fig 4C). These experiments showed that transient or perhaps pulsed exposure to doxycyclin is sufficient to achieve a good transiently induced knockdown of a chosen target protein. Such conditions may be desirable in experimental settings where the presence of doxycycline could be unwelcome, for example in combined knockdown/drug tests. To conclude these tests, we analyzed the transferability of the whole system to other MM cell lines which had also been stably transposed with the pT2-tetR vector (L-363, INA-6, OPM-2, MM.1S). We found that the effects seen for JJN-3 cells with respect to tetR-mediated shRNA expression, as well as doxycyclin-mediated induction of ERK2 knockdown, were reproducible across the board (Fig 4D).

**Fig 5:**
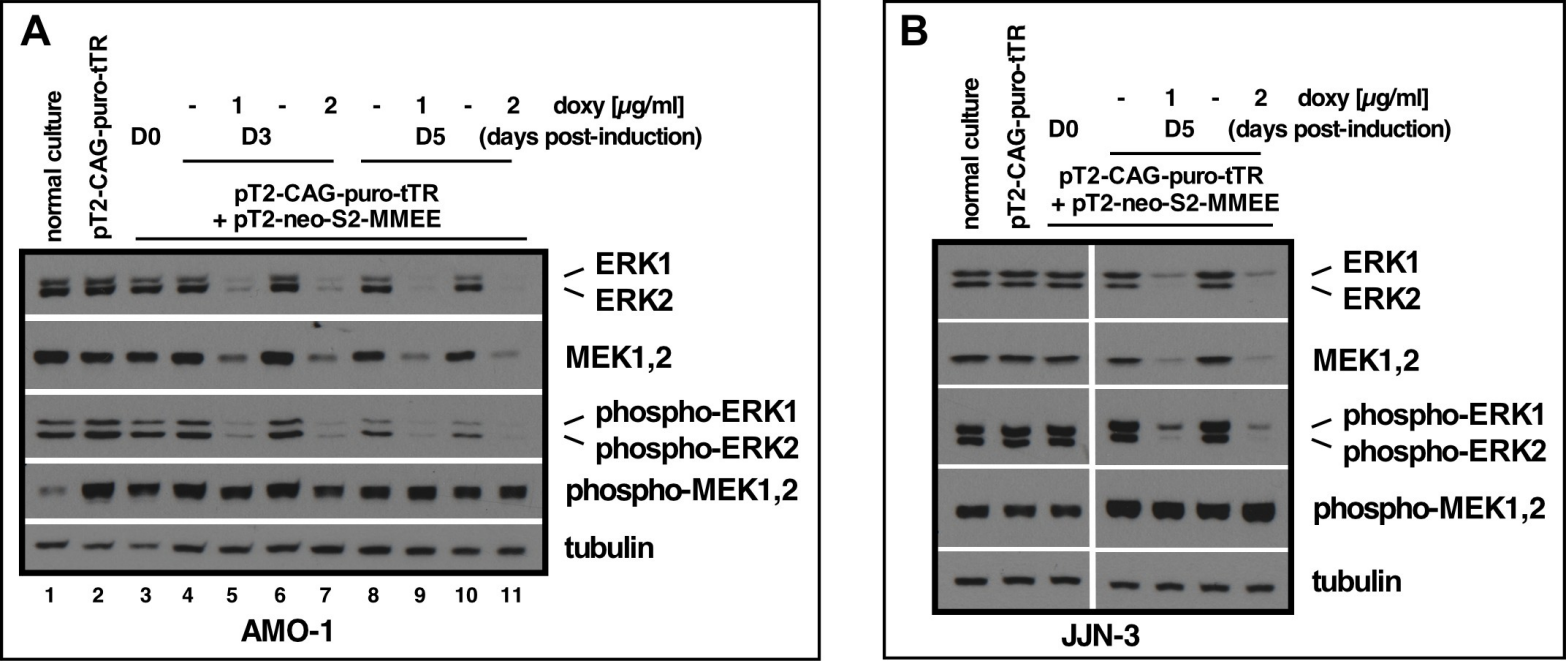
Multi-cassette induced knockdown of all 4 proteins of the MEK1/2-ERK1/2 module. (A) pT2-CAG-puro-tTR + pT2-neo-S2-shMEK2-shMEK1-shERK2-shERK1 (= pT2-neo-S2-MMEE) AMO-1 cells treated with either 1 or 2 μg/ml doxycyclin and harvested for Western blotting 3 and 5 days post-induction (D3/D5). D0 denotes the same cells harvested directly prior to doxycyclin treatment. (B) Similar experiment as in A but conducted with JJN-3 cells. Western blotting shown for cells harvested at day 5 post-induction with 1 or 2 μg/ml doxycyclin. All targets were stained on different blots, but with equal amounts of sample loaded from a single preparation. The respective tubulin loading controls are thus representative for all associated blots.

Finally, we tested a quadruple shRNA expression vector, in which each individual shRNA expression cassette is regulated via the 2x tetO elements (pS2-based). Transposition of this pT2-S2-shMEK2>shMEK1>shERK2>shERK1 vector (for simplicity named pT2-neo-S2-MMEE) into AMO-1-tetR cells followed by G418 selection for about 2 weeks yielded cells that in uninduced conditions showed no detectable depletion of ERK1 or ERK2, nor of the band that represents MEK1 and MEK2 when compared to normal AMO1 cells or the tetR-expressing subline (Fig 5A, lane 3 vs. lanes 1 and 2). Treatment with either 1 or 2 μg|ml doxycyclin and harvest of cells at 3 and 5 days post-induction, confirmed strong knockdown of all four targeted proteins. As had previously been observed for the single ERK2 knockdown, maximum effects were mostly achieved by day 3 and could then not significantly be increased. A similar experiment performed in JJN-3-tetR cells confirmed these results and also showed that the effects obtained after induced shRNA expression from the stably transposed quadruple expression vector were on a par with the results previously seen for the transient approach (Figs 2D and 5B).

In summary, we describe a simple vector system for the concatenization of any desired number of shRNA expression cassettes and their transposase-mediated transfer into mammalian cells. The shRNA expression cassettes can be used either for constitutive knockdowns if the recipient cells lack the tetR protein, or they can efficiently be repressed if the tetR protein is present. Doxycyclin-mediated induction in either temporal or constant manner can then induce the concerted knockdown of at least four different targets without apparent loss of efficiency, and without selection bias against noxious shRNAs during establishment of the stably transfected cell cultures. The system is cost-effective, reproducible and easy to implement, since it uses only simple cloning procedures and standard plasmid preparations, and requires just the most basic biosafely level. Although we have only tested these vectors in multiple myeloma cell lines these belong to the harder-to-transfect types of cell, and in our experience a transformed culture will emerge from the antibiotic selection as long as a few percent of strongly transfected cells (simply judged via addition of an expression plasmid for EGFP) can be produced.

Apart from addressing a number of different targets simultaneously this system could also be adapted for related approaches, such as the consecutive knockdown of different targets or of different splice forms of the same protein (if the coding mRNA forms can individually be addressed), by combining constitutively active and inducible shRNA expression cassettes within the same vector. It might also be possible to use this system to knock down recalcitrant proteins by a combination of different shRNA expression cassettes that target the same gene. However, while such an approach is conceptually easy to achieve, it would still rely on the availability of good target sequences. In principle, there is no limitation to the number of shRNA expression cassettes that can be assembled, although in practice there will be an (as yet untested) limit to the insert size of the vectors employed. Another potential limitation could be increasing interference between the identical units that form the multi-shRNA expression cassette block, although no sign of such an effect was visible here up to n = 4 units.

The shRNA target sequences used here for stable knockdown with multi-cassette vectors had all been verified as highly efficient in stringent transient transfection protocols, i.e. only the most strongly transfected cells had been specifically purified and knockdown efficiency was then assessed in this fraction [16]. Such a feature may well be imperative to achieve good knockdown levels from at best a few copies of stably integrated shRNA expression cassettes per cell. Good prior characterization of the shRNA sequences to be used is therefore essential. Another potential limitation of this system–compared to transient transfections with siRNA or shRNA expression vectors, and especially in comparison with small molecule inhibitors–is again related to the need for synthesis of the functional RNAi reagents from the low base of just one or a few stably integrated shRNA expression cassettes. After induction, cells may have enough time to counter the gradual loss of the target protein by compensation mechanisms, such as increased activity of the residual protein or co-option of provisions for redundancy. This needs to be evaluated on a target-by-target basis, but such effects may be more pronounced for (essential) catalytic target proteins (kinases etc.) than for structural proteins such as adaptor molecules or transcription factors.

Lastly, it should be mentioned that the use of the system as described here can fairly easily be adapted to work with different vector systems such as adenoviral or lentiviral vectors, if they are engineered to contain a single *Sac* I compatible cloning site to accept the shRNA expression cassette(s) construct. Furthermore, researchers who prefer the use of other pol III promoters, such as U6 or 7SK, could easily introduce the changes required to turn these into concatenizable constructs, either with or without induction properties, in order to keep working with a system that they know and might have found to best suit their specific research needs.

Note: The plasmids pSUS, pSUSTER2, pT2-NBneo and pT2-tetR, as well as their full sequence information, will be deposited with Addgene. All vectors mentioned in this paper are also available upon request.

## Supporting information

S1 File Vector sequences. (RTF)
